# Risk Adjustment in Health Insurance Markets: Do Not Overlook the “Real” Healthy

**DOI:** 10.1097/MLR.0000000000001955

**Published:** 2023-12-04

**Authors:** Richard C. van Kleef, René C.J.A. van Vliet, Michel Oskam

**Affiliations:** Erasmus School of Health Policy & Management, and The Erasmus Centre for Health Economics Rotterdam, Erasmus University, Rotterdam, The Netherlands

**Keywords:** health insurance, regulated competition, risk selection, risk adjustment

## Abstract

**Objectives:**

The goals of this paper are: (1) to identify groups of healthy people; and (2) to quantify the extent to which the Dutch risk adjustment (RA) model overpays insurers for these groups.

**Background:**

There have been strong signals that insurers in the Dutch regulated health insurance market engage in actions to attract healthy people. A potential explanation for this behavior is that the Dutch RA model overpays insurers for healthy people.

**Methods:**

We identify healthy groups using 3 types of ex-ante information (ie, information available before the start of the health insurance contract): administrative data on prior spending for specific health care services (N = 17 m), diagnoses from electronic patient records (N = 1.3 m), and health survey data (N = 457 k). In a second step, we calculate the under/overpayment for these groups under the Dutch RA model (version: 2021).

**Results:**

We distinguish eight groups of healthy people using various “identifiers.” Although the Dutch RA model substantially reduces the predictable profits that insurers face for these groups, significant profits remain. The mean per person overpayment ranges from 38 euros (people with hospital spending below the third quartile in each of 3 prior years) to 167 euros (those without any medical condition according to their electronic patient record).

**Conclusions:**

The Dutch RA model does not eliminate the profitability of healthy groups. The identifiers used for flagging these groups, however, seem inappropriate for serving as risk adjuster variables. An alternative way of exploiting these identifiers and eliminating the profitability of healthy groups is to estimate RA models with “constrained regression.”

Like many other countries, the Netherlands has organized its social health insurance scheme according to principles of “regulated competition.” Important aspects of competition include the option for consumers to annually switch insurance plans and the opportunity for insurers to apply managed care techniques, such as selective contracting of health care providers. The main aspects of regulation include an individual insurance mandate, standardization of the benefits package, open enrollment, premium-rate restrictions, and risk adjustment (RA). The goal of having regulated competition is to simultaneously achieve objectives related to fairness (eg, accessibility and affordability of coverage) and efficiency (eg, value for money).

The Dutch RA model is one of the most sophisticated RA models in the world. In addition to demographic variables, it contains a wide range of socioeconomic variables and morbidity classifications. Variables based on socioeconomic information include the source of income (eg, employed vs disability allowance), household income, level of education, household composition, and regional characteristics. Morbidity classifications include diagnostic cost groups (based on inpatient and outpatient hospital diagnoses in the prior year), pharmaceutical cost groups (based on prescribed drugs in the prior year), durable medical equipment cost groups (based on the use of medical equipment in the prior year), physiotherapy diagnoses cost groups (based on diagnoses reported by physiotherapists in the prior year), and multiple-year high cost groups (based on high spending in 3 prior years). The RA model is meant to compensate insurers for the predictable profits and losses that would otherwise result from premium-rate restrictions. The underlying idea is that in the absence of predictable profits and losses, insurers are incentivized to serve all consumers, including the sick. Over the past few years, however, there have been strong signals that insurers engage in selection activities to attract healthy people.^1–3^ A potential explanation for this behavior is that the RA model does not eliminate the profitability of healthy people.

The goal of this study is twofold: (1) to identify groups of healthy people; and (2) to quantify the extent to which the Dutch RA model overpays insurers for these groups. For this exercise, we exploit 3 types of data covering a broad spectrum of individual-level information: administrative data on spending for specific health care services (N = 17 m), diagnoses from electronic patient records (N = 1.3 m), and health survey information (N = 457 k). An important feature of our approach is that healthy groups are identified ex-ante with information that is available before the start of the health insurance contract. The motive for this approach is that we are interested in expectations of profitability, which potentially influence insurer behavior.

## METHODS

The starting point for our analysis is a dataset with individual-level information on health care spending and risk characteristics for nearly the entire Dutch population of 2017. Originally, these data came from various administrative sources, including insurers, the tax collector, and the registration service for social benefits. Spending includes all services covered by the Dutch basic health insurance, such as primary care, hospital care, and pharmaceutical care. Risk characteristics include all risk adjusters used in the RA model of 2021. This dataset allows replicating the Dutch RA model of 2021 on spending from 2017. By merging RA outcomes with external information on health, we can assess the under/overpayment for various (healthy) groups. Further, we describe our data and methods in more detail.

### Step 1: Identification of Healthy Groups

Our first objective was to identify groups that are relatively healthy at the start of the contract period, in this case: January 1, 2017. In an explorative run through the data, we searched for such groups from 3 different angles: (1) administrative data (eg, prior spending on specific types of care), (2) objective health (ie, diagnostic information from electronic patient records), and (3) subjective health (ie, self-reported health and limitations).

The administrative data include individual-level information on health care spending in the years 2014, 2015, 2016, and 2017. We used the spending data from 2014, 2015, and 2016 to identify several groups with “multiple-year low spending” for specific types of health care as an indicator of being relatively healthy in 2017. Two exemplary groups were selected for presentation in this paper: (1) people who remained below the third quartile of hospital spending (about 600 euros) in each of the 3 years and (2) people who remained below the third quartile of pharmaceutical spending (about 170 euro) in each of these years. The third quartile of spending was based on the entire population, including those with zero spending. “Hospital spending” includes both inpatient and outpatient hospital spending.

The dataset with diagnostic information from electronic patient records came from the Nivel Primary Care Database (Nivel-PCD). Nivel-PCD routinely collects data from electronic health record systems, including information about consultations, morbidity, prescriptions, and diagnostic tests, in a sample of about 400 general practices, including 1.3 million registered patients. In a preliminary analysis, we found that this sample is already reasonably representative of the entire Dutch population. Further (under step 2), we describe how we corrected for remaining differences between the sample and the population. Diagnoses in this dataset are coded according to the International Classification of Primary Care which distinguishes 685 diagnoses.^4^ For each of the 1.3 million registered patients, the dataset indicates whether a diagnosis was registered in 2016. In case of “chronic conditions,” diagnoses from the years 2014 and 2015 were used as well. In the total set of 685 diagnoses, Nivel labeled 109 health conditions “chronic” using the following rule of thumb: a disease is considered as chronic when it is unlikely that people who have been suffering from that disease will fully recover. For more information about Nivel-PCD, we refer to a study by Nielen et al.^4^ Based on this dataset—which we will hence refer to as “GP data,” with GP for General Practitioner—we identified 2 healthy groups: (1) people with none of the 685 diagnoses in 2016 and (2) people with none of the 109 chronic diagnoses in 2016. In light of this study, it is worth mentioning that GP patient records in the Netherlands are expected to give a complete overview of a person’s health status. First, in the Netherlands, all outpatient drug prescriptions are done by GPs. Second, the GP functions as a gatekeeper to specialized care, meaning that any person in need of specialized care will first have to see his GP to obtain a referral. Third, almost all Dutch inhabitants are registered with a GP. Because of these features, it is highly likely that when a person’s record does not make mention of a (chronic) medical condition, that person is indeed healthy.

The health survey information came from the Public Health Monitor that was conducted in 2016 by the Community Health Services in collaboration with Statistics Netherlands and the National Institute for Public Health and the Environment. This dataset includes information on self-reported general health (both physical and mental) and chronic conditions for 457 k respondents. For 3 reasons, the composition of the survey sample differs from that of the total population. First, the survey was not sent to people living in a long-term care facility. Second, the sample only includes people of 19 years or older. Third, some groups were—purposely—oversampled (eg, the elderly). To make the sample representative for the entire population of 19 or over, Statistics Netherlands reweighted the sample using the following characteristics: age, sex, marital status, degree of urbanization, household size, ethnicity, income, and region. Further (under step 2), we describe how we corrected for remaining differences relative to the total population of 19 or over. Based on the survey, we identified several healthy groups, of which the following 4 exemplary groups were selected for presentation in this paper: (1) people who reported to be in good or very good health in 2016, (2) people who reported no chronic condition in 2016, (3) people who reported no physical limitations in 2016 and (4) people with a low risk of mental disorders in 2016 (derived from questions on mental health).

### Step 2: Calculation of Group-Level Under/Overpayment

As a next step, we calculated RA outcomes for the healthy groups identified in step 1. For this exercise, we took the Dutch RA model of 2021 as a starting point. As described above, this model includes a broad set of risk adjusters. The RA system of 2021 consists of 3 separate prediction models, one for each of the following spending types: somatic care (covering 90% of spending in the 2017 data), mental care (covering 10% of spending in 2017 data), and out-of-pocket spending below the mandatory deductible of 385 euro. Each of these models generates a prediction that forms the basis for the RA payments to insurers. From the perspective of insurers, total predicted spending $\hat{y}$ for individual *i* is calculated as


$${\hat{y}}_{i,\mathrm{total}}={\hat{y}}_{i,\mathrm{somatic}}+{\hat{y}}_{i,\mathrm{mental}}-{\hat{y}}_{i,\mathrm{oop}}\;(1)$$

For a detailed description of the 3 RA models, their risk adjusters, and the estimation procedures, we refer to the study by Van Kleef et al.^5^ Specific details about the Dutch RA models for 2021 can be found in Staatscourant no. 17199.^6^ In our simulation analysis, we used formula (1) to calculate individual-level predicted insurer spending for 2017. In addition, analog to formula (1), we calculated actual insurer spending y for individual *i* as


$$y_{i,\mathrm{total}}=y_{i,\mathrm{somatic}}+y_{i,\mathrm{mental}}-y_{i,\mathrm{oop}}\;(2)$$

With formulas (1) and (2), we were able to calculate the mean financial result for group *g* as


$${\mathrm{Mean}\;\mathrm{Financial}\;\mathrm{Result}}_{g}=\frac{\sum_{i\in g}\left({\hat{y}}_{i,\mathrm{total}}-y_{i,\mathrm{total}}\right)}{n_{g}}\;(3)$$

A positive (negative) mean financial result for group *g* can be interpreted as overpayment (underpayment) of insurers for group *g*.

As mentioned previously, the GP sample (N = 1.3 m) is not perfectly representative of the entire Dutch population. To mitigate the remaining differences, we applied an iterative proportional fitting procedure using the risk adjuster classifications from the total sample. With this procedure, we generated a “weight” for each individual in the GP data.^7^ Using this weight in the analysis guarantees that mean spending and relative frequencies of risk adjustor flags in the GP sample equal those in the total population. With this procedure, it can be expected that the outcomes for groups identified in the GP sample are representative of the total population. In other words, it can be expected that, if the GP data had been available for the entire Dutch population, the mean financial result for healthy groups would have been similar to those found in the sample. We applied the same procedure to the survey sample (N = 457k), but then with the total population of 19 years or older as our reference point.

## RESULTS

Table 1 presents mean “insurer spending” for the healthy groups and the “complementary groups” identified in the administrative data and GP information. By “insurer spending” we mean all spending covered by the insurer (which excludes the out-of-pocket spending by consumers). By “complementary group” we mean the group of people that are not part of the healthy group given a specific definition. For all 4 groups mean insurer spending is substantially lower than in the complementary group, indicating that the 4 groups are indeed relatively healthy. Mean per person insurer spending in 2017 was lowest for the group “no condition reported by the GP in 2016” (548 euros). In terms of size, this group is relatively small: 11% of the total population. The other 3 groups are much bigger, with sizes ranging from 45% (no chronic condition reported by GP in 2016) to 65% (pharmaceutical spending <Q3 in 2016, 2015, and 2014).

**TABLE 1:** Healthy Groups Identified in the Administrative Data and Rebalanced GP Data (All Ages)

			Mean per person insurer spending in 2017 (euros)	
Source	Subgroup	Percentage of population	Healthy group	Complementary group
Administrative data (N = 17 m)	Hospital spending <Q3 in 2016, 2015, and 2014	53	981	3983
	Pharm. spending <Q3 in 2016, 2015, and 2014	65	976	4979
Diagnoses from GP (N = 1.3 m)	No condition reported by GP in 2016	11	548	2633
	No chronic condition reported by GP in 2016	45	961	3568

Mean per person insurer spending in the total population equals 2395 euros per person per year.

GP indicates general practitioner.

Table 2 shows the same information as Table 1, but this time for groups identified in the rebalanced survey information. As these groups are identified in the population of 19 years and older, overall insurer spending is higher than in Table 1 (see footnotes following Table 1 and 2). Nevertheless, the general pattern in Table 2 is similar to that in Table 1: mean insurer spending is much lower for the healthy groups than for their complements.

**TABLE 2:** Healthy Groups Identified in the Rebalanced Survey Data (19 years or older)

			Mean per person insurer spending in 2017 (euros)	
Source	Subgroup	Percentage of population 19+	Healthy group	Complementary group
Health survey (N = 457 k)	(Very) good self-reported health in 2016	74	1643	5865
	No self-reported condition in 2016	65	1431	5133
	No self-reported limitations in 2016	65	1422	5156
	Low risk of mental disorders in 2016	52	1991	3550

Mean per person insurer spending in the total population of 19+ equals 2744 euros per person per year.

**FIGURE 1:**
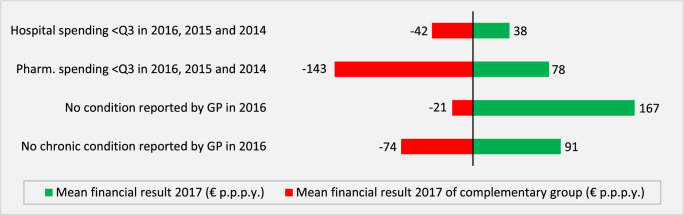
Mean per person financial result for healthy groups (green bars) and their complements (red bars) identified in the administrative data or rebalanced general practitioner data (all ages). All under/overpayments are statistically significantly different from zero (*P* < 0.01).

**FIGURE 2:**
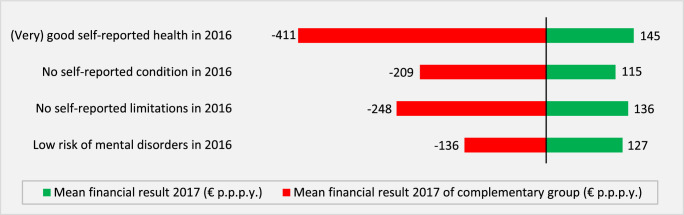
Mean per person financial result for healthy groups (green bars) and their complements (red bars) identified in the rebalanced survey data (19 years or older). All under/overpayments are statistically significantly different from zero (*P* < 0.01).

The results in Table 1 and 2 imply that—in a hypothetical situation of community-rated premiums and no RA—all 8 healthy groups would be highly profitable to insurers. For example, the unadjusted predictable profit for the group “no condition reported by GP in 2016” (Table 1) would be 1847 euros per person per year, that is, the difference between mean insurer spending in the total population (2398 euros) and the group of interest (548 euros). For the other groups in Table 1, the unadjusted predictable profit for insurers would vary from 1392 to 1701 euros per person per year, and for the groups in Table 2 from 404 to 973 euros per person per year. The key question is: “How much of these predictable profits remain when taking into account the Dutch RA model of 2021?” Figures 1 and 2 provide the answer. The green bars show the mean financial result “net” of RA for the healthy groups, whereas the red bars show the mean financial result “net” of RA for the complementary groups.

Figures 1 and 2 reveal a clear pattern: under the Dutch RA model of 2021, healthy groups remain profitable to insurers. Mean per person profits “net of RA,” however, are substantially smaller than the “unadjusted profits.” For example, the RA model reduces the profit for the group “no condition reported by GP in 2016” from 1847 to 167 euros per person per year, a decrease of 91%. For the other 7 groups, this reduction varies from 69% (low risk of mental disorders in 2016) to 97% (hospital spending <Q3 in 2016, 2015, and 2014).

## DISCUSSION

Our findings show that in the Dutch regulated health insurance market, healthy groups remain predictably profitable for insurers, despite sophisticated RA. For insurers, it makes a difference of—on average—165 euros per person per year whether they enroll an individual without a chronic disease (mean financial result = 91 euros per person per year) or an individual with a chronic disease (mean financial result = −74 euros per person per year). For the other partitions shown in Figures 1 and 2, this “absolute difference in mean financial result” ranges from 80 euros per person per year (yes/no “hospital spending <Q3 in 2016, 2015 and 2014”) to 556 euros per person per year (yes/no “good or very good self-reported health in 2016”). Given that the Dutch RA model is one of the most sophisticated RA models in the world, it could well be that RA models used in other countries generate overpayments for healthy people too. Whether this is indeed the case remains an empirical question.

A potential explanation for the finding that healthy groups are predictably profitable is that these groups are “overlooked” in RA research. Studies on the improvement of RA models typically focus on new/better ways to identify high-risk people. For example, by developing better risk adjusters for flagging diabetics, compensation for this patient group can be improved. Over the past decades, sophisticated disease classifications have been developed and refined. Examples include the DGGs,^8^ pharmaceutical cost groups,^9^ durable medical equipment cost groups,^10^ physiotherapy diagnoses cost groups,^11^ and multiple-year high cost groups^12^ applied in the Netherlands, but also the hierarchical condition categories,^13^ clinical risk groups,^14^ and chronic illness and disability payment system^15^ that have been applied in health insurance schemes in the United States. When used as risk adjusters, these classifications do not only reduce incentives for insurers to select against high-risk people but also reduce incentives for insurers to select in favor of the healthy. More specifically, these risk adjusters increase RA payments for people with a morbidity flag and decrease RA payments for people without a morbidity flag. Nevertheless, groups of healthy people are likely to remain profitable to insurers. The reason is that disease classifications tend to be incomplete. For use in disease classifications, information must fulfill specific criteria related to incentives and feasibility.^16,17^ Some information might be predictive of health care spending, but inappropriate for use in disease classifications. Examples include health indicators that would introduce incentives for gaming and indicators that are not available for the entire population of interest. When such indicators are excluded, however, the resulting disease classifications may not identify the entire group of people with a chronic disease. Consequently, the group without a disease flag does not only comprise the healthy people but also some of the unhealthy ones. While the RA model will compensate for the difference in mean spending between the groups with and without a disease flag, the “real” healthy are likely to remain predictably profitable.

An important question for health policy and research is “How to further reduce the predictable profits on the real healthy?” The conventional method for eliminating the predictable profit/loss for group *g* is to include an indicator for *g* in the RA model. In the case of the healthy groups identified in our analysis, however, this strategy might be problematic as the “identifiers” do probably not fulfill the criteria for risk adjusters related to incentives and feasibility. For example, when used as a risk adjuster, the identifiers “hospital spending <Q3 (about 600 euros) in 2016, 2015, and 2014” and “pharmaceutical spending <Q3 (about 170 euros) in 2016, 2015, and 2014” would lead to perverse incentives. More specifically, when hospital spending for an enrollee exceeds 600 euros this year and/or pharmaceutical spending exceeds 170 euros this year, the insurer will receive a higher RA payment in the next 3 years. Such a link between spending and RA payment reduces incentives for insurers to control costs and could even provide them with incentives for pushing enrollees over the cost threshold, depending on the payment weight for these risk adjusters.^19^ Recent literature shows that insurers are indeed responsive to perverse incentives generated by RA models.^19,20^ The other groups identified in this paper are inappropriate for serving as risk adjusters too, as the data used for identification (ie, the electronic patient records and health survey data) is not available for the entire population, which is a requirement for RA models that use individual-level risk adjusters.

To the extent that identifiers of healthy groups are considered inappropriate for serving as a risk adjuster, regulators could consider an alternative solution to exploit the predictiveness of these identifiers, constrained regression. This method allows for imposing so-called “constraints” on the estimated payment weights. An example of such a constraint is that the overpayment for a healthy group equals zero. For applications of this method in RA models, we refer to the studies by Van Kleef et al,^21^ McGuire et al,^22^ and Withagen-Koster et al.^23^ Van Kleef et al^21^ have used constrained regression to eliminate under/overpayments for groups identified with information on prior spending. The authors show how constrained regression can eliminate under/overpayments for these groups without introducing the perverse incentives that would occur when prior spending was used as a risk adjuster. In the same spirit, McGuire et al^22^ have applied constrained regression as an alternative for risk adjuster variables that are potentially vulnerable to gaming. Withagen-Koster et al^23^ have used constrained regression to eliminate under/overpayments for groups identified with survey information. The authors show how constrained regression can eliminate under/overpayments for these groups without having the identifiers (survey data) available for the entire population. All 3 studies demonstrate how constrained regression can be a powerful tool for shifting RA funds from healthy groups to unhealthy groups. For example, Van Kleef et al^21^ and McGuire et al,^22^ show that “constraints” aimed at reducing the underpayment of unhealthy groups also reduce the overpayment of healthy groups.

Further reduction of predictable profits and losses is crucial for improving the functioning of health care systems. A growing literature shows that insurers respond to these predictable profits and losses by engaging in risk selection.^2,24–28^ For example, Stolper et al^2^ have found that in the Dutch health insurance market, insurers with different brands primarily use their subbrands as strategic vehicles to improve their competitive positions by targeting these brands at financially favorable groups and price sensitive buyers. In general, risk selection threatens the functioning of health insurance systems as it can result in suboptimal design of insurance plans.^29^ It can also lead to suboptimal sorting of consumers into plans as premium differences among plans might not only reflect variation in efficiency (value for money) but also variation in health across portfolios.^30^ Moreover, selection-driven premium differences can conflict with fairness objectives, for example, when society prefers financial contributions to health care being independent of health risk.

## CONCLUSION

In sum, we conclude that even sophisticated RA models might not eliminate the predictable profits for healthy people. A potential explanation is that the “real” healthy are overlooked in RA policy and research. Further improvements of RA models are needed to eliminate the profitability of healthy people and improve the functioning of health insurance markets.
